# The relationship of exercise and cancer-related fatigue in patients with advanced liver cancer: a cross-sectional study

**DOI:** 10.1038/s41598-023-44655-w

**Published:** 2023-10-13

**Authors:** Juan Li, Qinqin Cheng, Xiangqian Zhu, Sha Lin, Huan Xiang, Wen Lu

**Affiliations:** Hunan Cancer Hospital, Changsha, Hunan China

**Keywords:** Cancer, Health care, Physics

## Abstract

There is increasing interest in understanding exercise as a potential treatment for cancer-related fatigue (CRF); however, rarely research has been conducted on more aggressive cancers with short survival, such as liver cancer. The purpose of this study was to provide educational ideas for insufficient exercise and provide clues for the design of effective and safe exercise intervention programs with high compliance in patients of advanced liver cancer in the future. Participants were recruited from a tertiary cancer hospital using convenience sampling. All participants were asked to complete self-report questionnaires that assessed their medical and demographic variables, exercise habits and CRF during their hospitalization in the interventional department. Spearman’s correlation analysis and Nonparametric test was used to explore correlations between exercise subgroups and CRF. The Baron and Kenny’s Approach was used to investigate the mediating effect of exercise index between P-EX and CRF. 207 out of 255 participants were enrolled in this study, with an average age of 55.4 years. The CRF score was 33 (28, 36), and 93.2% had insufficient exercise. Exercise frequency (≥ 3 Times/week) (Z = 4.34, p = 0.037) and maintaining exercise trend (Z = 15.85, p = 0.001) had a positive effect on CRF. P-EX had a great impact on exercise index and affecting CRF directly. Participants in the study showed serious fatigue and insufficient exercise. Exercise education can be initiated earlier, particularly those without regular exercise experience. Sustained light exercise, compliant with exercise habits and interests, three times a week may be a practical way to reduce the risk of CRF in advanced liver cancer.

## Introduction

Liver cancer was the sixth most commonly diagnosed cancer and the third leading cause of cancer-related deaths worldwide in 2020, with approximately 906,000 new cases and 830,000 deaths^1^. Liver cancer, which is characterized by high incidence, high mortality, and poor prognosis, has a high symptom burden^2,3^. Cancer-related fatigue (CRF) rates are up to 85%, of which 9 to 45% report moderate-to-severe CRF, and 21 to 52% of survivors still experience CRF for up to 3 years post-diagnosis^4–6^. The National Comprehensive Cancer Network describes CRF as “a painful, persistent, and subjective sense tiredness or exhaustion related to cancer or cancer treatment^7^. CRF has a seriously negative impact on physical function, psychology, emotion, cognitive^8,9^ and is associated with higher mortality^10^. Most doctors and nurses in developing countries lack a strong understanding of CRF, greatly underestimate its prevalence and harmfulness, and feel helpless about improving CRF.

As far as we know, there is no standard intervention for CRF. Based on the current evidence^11–13^, although pharmacological treatments have been used to prevent or improve CRF, compared with exercise interventions, including only those planned, structured, and repetitive activities aimed at improving or maintaining one or more components of physical fitness, they are the least effective for CRF during and after treatment. The American College of Sport Medicine guidelines for exercise in cancer published in 2018 suggests that moderate-intensity aerobic or resistance or a combination training can significantly reduce CRF^14^. Moreover, patients who exercised before and after a cancer diagnosis were observed to have a lower relative risk of cancer mortality and recurrence and experienced fewer/less severe adverse effects^15^. However, most studies^16,17^were focused on patients with low tumor invasion and symptom burden, such as breast cancer, prostate cancer, while exercise attention was rare in more aggressive cancers with short survival times, including pancreatic and advanced liver cancers. Up to date, although growing evidence supports the safety and efficacy of exercise, no universal recommendation or specific exercise guidelines for patients with liver cancer are available.

Historically, patients with cancer were advised to rest, recover, and save energy, avoiding engaging in tiring exercise. Exercise deficiency is widespread in many countries worldwide; 40–93% of patients with cancer lack exercise^18–20^, and the amount of exercise decreased sharply and even no exercise at all after cancer diagnosis^21^. A low adherence and high drop-out rate were reported in many intervention studies^22,23^. Engaging in exercise programs is particularly arduous for patients with more aggressive cancers, mainly because of a series of physical and psychological disease-related barriers. The first randomized clinical trial of resistance training in patients with pancreatic cancer by Karen Steindorf and colleagues indicated that resistance training is safe for patients and improves physical function, sleep problems and CRF; however, recruitment and compliance with exercise are unsatisfactory^24^.

The theory of planned behavior^25^ proposes current behavior is strongly influenced by past behavior. We hypothesized that P-EX (Past exercise, self-reported exercise before diagnosing cancer) behavior habits can affect present behavior patterns and exercise compliance, thus affecting CRF. Therefore, understanding the exercisehabits of patients and analyzing the influence of different subgroups such as exercise time, exercise intensity and exercise trend on fatigue can not only provide ideas for the education program of insufficient exercise, but also provide suggestion for the design of exercise intervention program to ensure the compliance, effectiveness and safety in patients with advanced liver cancer in the future.

In this article, we described exercise behavior (time, frequency, intensity, the level and the trend), to understand the exercise-habits of advanced liver cancer. Exercise was defined as P-EX and the exercise index (self-reported exercise in the last week before their hospitalization). Moreover, we analyzed the relationship between exercise subgroups and CRF to provide educational ideas for insufficient exercise and provide clues for the design of effective and safe exercise intervention programs with high compliance in patients of advanced liver cancer in the future. This study could improve the specificity of the sports oncology literature to serve a wider range of cancer types and stages.

## Methods

### Participants

This cross-sectional survey was conducted in the Department of Tumor Intervention of a Tertiary Cancer Hospital in Hunan from April 2022 to October 2022. This study was reviewed and approved by the Ethics Committee for Clinical Trials (Prot. No. SBQLL-2021-174), Hunan cancer hospital. The liver cancer patients who take interventional operation were in the advanced stage of liver cancer, and lost the chance of surgery. A research nurse selected patients according to predefined inclusion and exclusion criteria. Before the investigation, patients were received a leaflet describing the purpose of the study and asked to provide signed informed consent. The study nurses contacted 255 hospitalized patients, 33 patients refused, 10 patients gave up filling in the questionnaire halfway and 5 patients checked the same option in all the questionnaires. Overall, 207 participants were included in this study. Survivors were eligible if they were (1) diagnosed with liver cancer by pathology; (2) undergoing interventional therapy or were about to undergo interventional therapy after discussion with the medical team; (3) aged ≥ 18 years; (4) conscious and able to communicate orally. The exclusion criteria were as follows: (1) They have diseases of the nerves, muscles, bones and joints that prevent exercise; (2) They have serious heart disease, severe high blood pressure and respiratory problems.

#### Sample size

Based on correlation analysis in cross-sectional studies, we estimated the required sample size as 20 times the number of variables. The study included 7 independent variables, and we expanded the calculated sample size by 20% to control for potential missing data, resulting in 168 participants as the minimum number required.

### Patient-reported assessments

A nurse administered paper-based questionnaires at the hospital. She was available in case the participants required help. After the patients completed the questionnaire, the investigator carefully assessed whether the items were completed properly and ensured no missing data.

#### Exercise index

Exercise index was evaluated using the leisure score index of the Godin leisure-time exercise questionnaire (GLTEQ), which is widely used for patients with cancer^26^. The GLTEQ inquires about the previous week’s leisure time frequency (times/week) of vigorous (fast running in heart rate, including vigorous swimming and playing basketball), moderate (a slight increase in heart rate, including fast walking and square dancing), and mild-intensity (normal heart rates, such as walking, yoga, and Tai Chi). In order to exclude the effect of hospitalization, we asked patients to self-report their exercise in the last week before their hospitalization. The exercise index, which measures the total amount of exercise, was calculated according to the following formula: exercise index = (9 × vigorous intensity frequency/week) + (5 × moderate intensity frequency/week) + (3 × mild intensity frequency/week). Patients are classified as active (exercise- Index ≥ 24) or insufficiently active (exercise- Index < 24) according to the 2010 release of the American College of Sports Medicine Exercise Guidelines for patients with cancer^27^.

P-EX and exercise trend (Remaining exercise, Decreased exercise, Increase exercise, and Maintained no exercise) were assessed using the following questions: “Before diagnosing cancer, did you practice regular exercise (exercise three or more times a week for more than 30 minutes each time for more than 3 months)? (Yes; No) and “Since the diagnosis of your illness, have you modified your exercise? (More than before; Less than before; Completely stopped; No).

### Cancer-related fatigue

Okuyama developed the cancer fatigue scale (CFS)^28^ in 2000 to evaluate CRF symptoms in patients with cancer. The scale comprises 15 items subscales as follows: physical, emotional, and cognitive. Each item is scored on a 5-point Likert scale. From “nothing at all” to “very much,” the score is 0–4, and the total score is 0–60. Additionally, higher scores indicate greater fatigue. The Cronbach’s alpha coefficient for this sample was 0.764.

### Demographic and clinical variables

Participants’ demographic and medical variables were assessed. Demographic variables included sex, age at diagnosis, educational level, marital status, family income, economic pressure, working status, payment of medical expenses, and Body-mass index. Medical variables included cancer type, comorbidities, the number of interventions (times), interval to the last intervention, liver function grading, pain, time from diagnosis, cancer staging, and anemia.

### Statistical methods

The double-entry principle was adopted to ensure accuracy. Data analyses were performed using SPSS version 25.0. Demographic and medical variables are described as frequency and proportion. Descriptive statistics were applied to summarize the demographic characteristics and clinic characteristics of the participants, and their CRF score, and exercise index. The associations of exercise variables (exercise frequency, exercise time, exercise intensity, exercise activity, exercise trend) with CRF were explored using the Nonparametric analyses. Spearman’s correlation analysis was used to explore correlations between exercise index and CRF. The Baron and Kenny’s Approach (B-K method) to investigate the mediating effect of exercise index between P-EX and CRF. Because the number of missing values for these variates was low (< 2%), we used complete cases. Statistical significance was set at p < 0.05.

### Ethics approval

This study was performed in line with the principles of the Declaration of Helsinki. Approval was granted by the Ethics Committee of Hunan Cancer Hospital (Date: 2021-8-25/ No. SBQLL-2021-174).

### Consent to participate

Informed consent was obtained from all individual participants included in the study.

## Results

A total of 222 questionnaires were collected. After eliminating incomplete questionnaires and those with obvious contradictions in contents, 207 were ultimately included, with an effective completion rate of 93.2%. The CFS score was 33 (28, 36). Table 1 presents demographic and clinical characteristics, CRF scores, and exercise index. The mean age was 55.4 years, and 62.3% (n = 129) were under the age of 59. Most of the participants (69.1%, n = 143) were in elementary to middle school, 54.6% (n = 113) of health insurance were rural cooperative medical care or own expenses. Up to 72.5% (n = 150) reported Grade C and 76.3% (n = 158) were primary liver cancer.

**Table 1:** Demographic and clinic characteristics, description of CRF score, and exercise index (N = 207).

Characteristics	Frequency N (%)		Exercise index (M (P25, P75))	CRF score (M (P25, P75))
Age (years)				
≤ 59	129 (62.3)		9 (0, 15)	33 (29, 36)
60–69	62 (30.0)		12 (0, 18)	32.5 (25.75, 35)
≥ 70	16 (7.7)		7.5 (0, 12)	33.5 (26.75, 41.75)
Gender				
Male	163 (78.7)		9 (0, 15)	32 (27, 35)
Female	44 (21.3)		9 (0.75, 15)	34.5 (32, 39)
Education level				
≤ Primary school	35 (16.9)		0 (0, 12)	34 (32, 38)
Junior high school	108 (52.2)		12 (3, 18)	33 (29, 35)
College or high school	47 (22.7)		9 (0, 12.75)	33 (26, 38)
≥ University	17 (8.2)		3 (0, 10.5)	33 (26, 42.5)
Marital status				
Unmarried	16 (7.7)		7.5 (1.25, 9)	32 (27, 36)
Married	191 (92.3)		9 (0, 15)	33 (29, 37)
Family income (CNY monthly)				
< 5000	153 (73.9)		9 (0, 15)	33 (29, 37)
5000–10,000	35 (16.9)		11 (4.5, 21)	29 (25.25, 35)
10,000–20,000	14 (6.8)		9 (0, 12)	34 (29.5, 37.5)
> 20,000	5 (2.4)		0 (0, 18)	34 (18, -)
Economic burden				
Very serious	94 (45.4)		9 (0, 18)	34 (32, 38)
Heavier	65 (31.4)		9 (0, 15)	31 (26, 36)
Commonly	39 (18.8)		12 (3, 15)	32 (26, 34)
Lighter	9 (4.3)		0 (0, 15)	32 (15, 31.5)
Working status				
Individual	13 (6.3)		10 (5.5, 15)	33.5 (26.5, 41.75)
Worker	4722.7)		6 (0, 15)	33 (29, 36)
Government agent	14 (6.8)		9 (0, 18)	30 (21.5, 33)
Farmer	133 (64.2)		9 (0, 16)	33 (27, 35)
Payment of medical expenses				
Urban residence	56 (27.1)		13.5 (4.5, 18.75)	33 (28, 35.5)
Provincial medical insurance	8 (3.9)		0 (0, 9)	35 (30, 48)
Municipal medical insurance	30 (14.5)		5.5 (0,12)	39.5 (26, 36.75)
RCMC	100 (48.3)		9 (0, 15)	33 (29, 37)
Own expense	13 (6.3)		6 (0, 9)	32.5 (29, 33.75)
Body-mass index(kg/m^2^)				
< 18.5	16 (7.8)		5 (0, 15)	37 (29, 45)
18.5–24.9	14 (69.4)		9 (0, 15)	33 (29, 37)
25–29.9	43 (20.4)		9 (0, 15)	32 (27, 34)
> 29.9	4 (1.9)		3 (0, –)	19 (11, –)
Cancer type				
Primary liver cancer	158 (76.3)		9 (0, 15)	33 (28.75,36)
Metastatic liver cancer	49 (23.7)		9 (0, 15)	32 (27,36.5)
Comorbidities				
Yes	59 (28.5)		9 (0, 15)	32 (29, 36)
No	148 (71.5)		9 (0, 15)	33 (27,36)
Times of interventions				
0	60 (29.0)		9 (0, 18)	32 (26.25, 32)
1	84 (40.6)		9 (0, 18)	32.5 (28, 35.5)
2–3	28 (23.2)		7.5 (0,12)	33.5 (29.25, 38.75)
> 3	15 (7.2)		9 (3, 15)	30 (26, 38)
Interval last intervention				
< 7 days	37 (25.2)		9 (1.5, 12)	33 (26, 35)
7 days to 1 month	34 (23.1)		9 (0, 15.75)	33.5 (29, 35.25)
2–6 months	55 (37.4)		6 (0, 15)	33 (22.5, 41.5)
> 6 months	21 (14.3)		12 (3,15)	32 (30, 34)
Liver function grading (Child–Pugh)				
Child-A	165 (79.7)		9 (2.25, 18)	33 (28, 36)
Child-B	42 (20.3)		3 (0, 11)	34 (29, 38)
Pain				
0	129 (62.3)		9 (0, 16.5)	33 (26, 36.5)
1–2	60 (29.0)		9 (0, 15)	33 (30, 37)
≥ 3	18 (8.7)		7.5 (0, 18)	34 (29.25, 42)
Time from diagnosis(years)				
< 1	129 (62.3)		9 (0, 18)	33 (29, 36)
1–2	44 (21.3)		9 (0, 15)	33 (25.5, 36)
2–3	16 (7.7)		10.5 (0.75, 15)	35 (32, 43.5)
> 3	18 (8.7)		11 (0, 12.75)	31 (26, 36.5)
Cancer staging (BCLC)				
B	61(29.5)		9 (0,18.5)	32.5 (23.75, 34)
C	150 (72.5)		9 (0, 15)	33 (29, 37)
Missing	2 (1.0)			
Anemia (HB)				
No (≥ 120 g/L)	171 (82.6)		9 (3, 15)	33 (28, 36)
Mild (90–119 g/L)	26 (12.6)		0 (0, 12)	35 (30, 41)
Moderate (60–89 g/L)	8 (3.9)		9 (0, 15)	35 (32, 40)
Heavy (< 60 g/L)	1 (0.5)			
Missing	1 (0.5)			

*RCMC* rural cooperative medical care. Child–Pugh grading criteria for the quantitative evaluation of liver reserve function in patients with liver cancer are commonly used in clinics. Grades A, B, and C indicated liver damage of varying severity (the higher the score, the worse the liver reserve function). *BCLC* barcelona clinic liver cancer, a clinical staging system for liver cancer. A (early), B (middle), and C (late). Comorbidities include high blood pressure, diabetes, heart disease.

In this survey, the exercise index of the participants was 9 (0, 15), with the highest being 63. exercise index was significantly correlated with CRF (r = 0.214 p = 0.002). Only 6.8% of the participants met the requirements for exercise and 30.4% exercised one or two times per week, 26.6% exercised for less than 30 min, and 77.8% exercised for light exercise. About 45% of participants increased or at least maintained exercise after diagnosis, whereas 35.3% gave up exercise and 19.3% reduced after a cancer diagnosis. exercise frequency (≥ 3 Times/week) (Z = 4.34, p = 0.037) and maintaining exercise trend (Z = 15.85, p < 0.001) had a positive effect on CRF in these patients, but exercise time, exercise intensity and exercise activity did not differ in their effects on CRF. Table 2 presents the relationship between physical activity variables and CRF.

**Table 2:** The relationship between exercise variables and CRF (N = 207).

Variable	Frequency (%)	Score (M (P25, P75))	Z	p-value
Exercise frequency (times/week)			4.34	0.037
1–2	63 (30.4)	33 (29, 37)		
≥ 3	124 (59.9)	32 (26.25, 34)		
Exercise time (min/time)			0.954	0.329
10–25 min	55 (26.6)	32 (27, 37)		
≥ 30 min	118 (57.0)	32 (26, 34)		
Exercise intensity			1.64	0.44
Mild	161 (77.8)	32 (27, 35)		
Moderate	20 (9.7)	31 (26.75, 34)		
Heavy	10 (4.8)	33.5 (31.25, 33.5)		
Exercise activity			0.11	0.739
Inactive	193 (93.2)	33 (28, 36)		
Active	14 (6.8)	31.5 (25, 37)		
Exercise trend			15.85	< 0.001
Maintaining	82 (39.6)	31 (25.75, 34)		
Reducing	40 (19.3)	32.5 (26, 38)		
Never	73 (35.3)	34 (30.5, 38)		
Improving	12 (5.8)	33 (29.5, 36.5)		
P-EX			12.77	< 0.001
Yes	122 (58.9)	32 (26, 34)		
No	85 (41.1)	34 (30.5, 38)		

*Exercise activity* active (exercise index ≥ 24) or insufficiently active (exercise index < 24). *P-EX* past exercise, self-reported exercise before diagnosing cancer.

**Figure 1:**
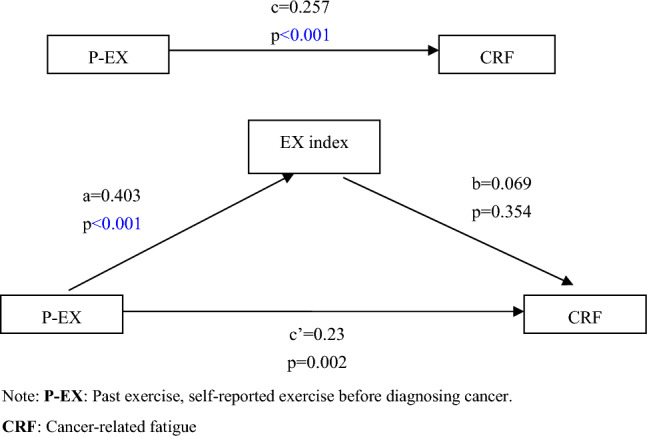
B-K test of the mediating effect of exercise index between P-EX and CRF. *P-EX* past exercise, self-reported exercise before diagnosing cancer, *CRF* cancer-related fatigue.

Figure 1 shows the association between exercise index, P-EX and CRF. The B-K mediation effect analysis showed that the total effect of P-EX on CRF was 0.257 (p < 0.001), and the standardized regression coefficient of P-EX on the exercise index was 0.403 (p < 0.001). After controlling the exercise index, the direct effect of P-EX on CRF was 0.23 (p = 0.002). However, the standardized regression coefficient of the exercise index on CRF was 0.069 (p > 0.05), indicating that exercise index does not have a mediating effect in the P-EX affecting CRF path.

## Discussion

To the best of our knowledge, this was the first study to describe the level and trend of exercise before and after diagnosis and to analyze the relationship between exercise and CRF in liver cancer. The purpose of this study was to provide educational ideas for insufficient exercise and provide clues for the design of effective and safe exercise intervention programs with high compliance in patients of advanced liver cancer in the future.

The fatigue score of the patients in this study was significantly higher than that in Okuyama’s^28^ study on patients with disease-free breast cancer. Compared with patients who have undergone successful surgical treatment, those with liver cancer may experience severe fatigue due to the disease’s characteristics and treatment. There was also a high correlation between exercise index and CRF in patients with liver cancer, and exercise might influence CRF by increasing lean muscle mass, aerobic capacity, and anti-inflammatory effects^29^.

We found that most of patients had regular exercise before diagnosis; however, after diagnosis, 93.2% of the participants (n = 193) had insufficient exercise, and 19.3% (n = 40) had obviously reduced or stopped exercise. This phenomenon hinted that many patients were at least aware of the benefits of sports activities but encountered different obstacles in the process of changing their behavior after receiving treatment. There were some evidences from other studies that insufficient exercise was widespread, and many survivors, who were offered a supervised exercise program, did not complete it^30–32^. In our report, the proportion of cancer survivors with insufficient exercise was higher than that reported in previous studies among cancer survivors^18,30^. This difference might be related to the fact that our participants had advanced liver cancer and were undergoing or about to undergo interventional surgery. Both physical and psychological stress caused by disease features with poor prognosis and treatment modalities with high symptom burden had a more severe negative impact on exercise. On the other hand, most participants came from rural areas with limited sports support conditions and lacked access to scientific exercise knowledge. They were threatened by the conventional belief that they should limit their activities, reduce their energy consumption, and rely on others to complete the activities needed in daily life once they feel tired. In summary, lack of awareness of scientific exercise, lack of access to planned services or lack of interest are important factors for exercise deficiency, and patient health status, disease burden and treatment methods are closely related. Therefore, more measures, including clinical education and research, should be taken to improve the knowledge and management of exercise in patients. Hospitals and communities should properly conduct supervised sports interventions, strengthen the establishment of rehabilitation sites for patients with cancer, and provide suitable sports sites for discharged patients.

We found that P-EX, which had a great effect on exercise index, had a direct effect on reducing fatigue, and the mediating effect of exercise index was not statistically significant. So, considering the impact of P-EX on exercise index and fatigue, we suggest the propaganda of exercise should be started early and throughout the disease, particularly those who had no exercise habits, to improve the awareness of the whole population. Several clinical studies have shown that exercise intervention in the preoperative stage or exercise of a longer duration can significantly affect CRF^33,34^. When designing the intervention of exercise plan, following patients’ exercise preferences and habits is an important factor affecting participation and consistency over time in a physical activity program^35^. However, there is a paucity of high-quality longitudinal studies that use objective measures to assess the standing effect of exercise in patients with cancer.

In addition, in the analysis of the correlation between exercise subgroup and CRF, we found an interesting result that the effect of exercise frequency on fatigue was different between groups, and the effect of exercise time, exercise intensity and exercise activity on CRF were not different between groups. It indicated that patients still need to exercise three times a week, but we may not pay too much attention to the amount of exercise, exercise intensity and exercise time while encouraging patients to exercise. A unified goal could be difficult to achieve for poor health status patients. For this reason, the exercise program may be flexible according to the patient’s physical condition. We may be able to adjust the time or intensity of exercise and to reduce the amount of exercise, even low levels of physical stretching, during treatment or when the symptom burden heavier^36^. In fact, the majority of participants in our study reported low-intensity exercise, and they preferred to choose forms of exercise that were simple, economical, and convenient (did not require expensive equipment, special skills, or tools, and could be performed both indoors and outdoors), such as walking, stretching. Spence showed that it was appropriate for patients with cancer to decrease their exercise during treatment and increase it slightly after treatment^37^. More consideration may be given to light exercise research in liver cancers with high tumor invasion and symptom burden. Guidelines primarily discuss light exercise to promote activity in people with low fitness^38–40^, based on the assumption that light exercise might act through similar paths as moderate-to-vigorous. Furthermore, Subgroup analysis of exercise trend revealed that maintaining exercise had a more positive impact on CRF than reducing exercise, while reducing exercise was greater than improving exercise. A randomized controlled trial which reported 4 years of follow-up after an 18-week supervised intervention in patients with breast and colon cancer showed that the positive effects of exercise could be maintained in the long term^41^. But, assessment of the long-term adherence and efficacy of exercise, post-intervention, is hampered by the generally short period of follow-up. The key is to encourage people to participate in and maintain the exercise. How to improve patients’ exercise compliance and persistence is an important consideration in designing exercise programs for patients with advanced cancer.

### Strengths and limitations

The strengths of this study are that it provided a detailed description of exercise habits and an analysis of the relationship between exercise subgroups and CRF in patients with liver cancer. It presented exercise educational ideas for service providers, as well as recommendations for researchers to improve the design of fatigue intervention measure in patients with high tumor invasion and high symptom burden.

However, this study also had some limitations. First, it was a single-center cross-sectional survey, and the data were self-reported with recall bias and lacked the statistical power to detect the influence efficiency of CRF and exercise. Second, selection bias may occur because patients with severe fatigue may be unwilling or unable to participate, and those without fatigue may not be interested in participating because they are not affected by this problem. Therefore, future studies should use replicated, objectively measured intervention studies to confirm the effect of mild exercise on CRF in liver cancer.

In conclusion, this study provided a clear understanding of exercise habits and the association between exercise and CRF in patients with advanced liver cancer. exercise education can be initiated earlier for liver cancer survivors particularly those without regular exercise experience. When designing exercise prescription, sustained light exercise three times a week, compliant with exercise habits and interests (e.g. walking), and with better flexibility and individuation may be a practical way to improve the patient’s exercise behavior and to reduce the risk of CRF in liver advanced cancer.

## Data Availability

The datasets used and analyzed during the current study available from the corresponding author on reasonable request.
